# A Multimodal Approach to the Early Treatment of a Facial Scar: A Case Report

**DOI:** 10.1002/ccr3.70719

**Published:** 2025-07-30

**Authors:** Jennifer Akl, Hamad El Hajj, Dima Jeha, Maya Habre

**Affiliations:** ^1^ Department of Dermatology Faculty of Medicine, University of Balamand Koura Lebanon; ^2^ Department of Dermatology Faculty of Medicine, Saint Georges Hospital University Medical Center, Saint George University of Beirut Beirut Lebanon

**Keywords:** botulinum toxin A, early wound intervention, facial scarring, laser

## Abstract

Facial scarring is associated with considerable psychosocial distress and can adversely affect overall quality of life. The combination of intralesional botulinum toxin injection and laser treatments in the early stage of wound healing markedly enhances scar appearance and improves cosmetic and psychological outcomes.

**Abbreviations**
BTX‐A: Botulinum toxin type A
cm: Centimeter
CO_2_
: Carbon dioxide
Er:YAG: Erbium‐doped Yttrium Aluminum Garnet
Hz: Hertz
J: Joule
ms: Millisecond
Nd:YAG: Neodymium‐doped Yttrium Aluminum Garnet
nm: Nanometer

## 1 Introduction

Wound healing is a multistep and complex process that leads to scar formation, resulting in physical, psychological, and aesthetic concerns. Therefore, treating wounds properly can help improve such inevitable consequences [1].

The combination of early postoperative interventions (laser, intralesional botulinum toxin, and topical antiscarring drugs) could aid patients in achieving an optimal and better cosmetic appearance [2].

## 2 Case History/Examination

**FIGURE 1:**
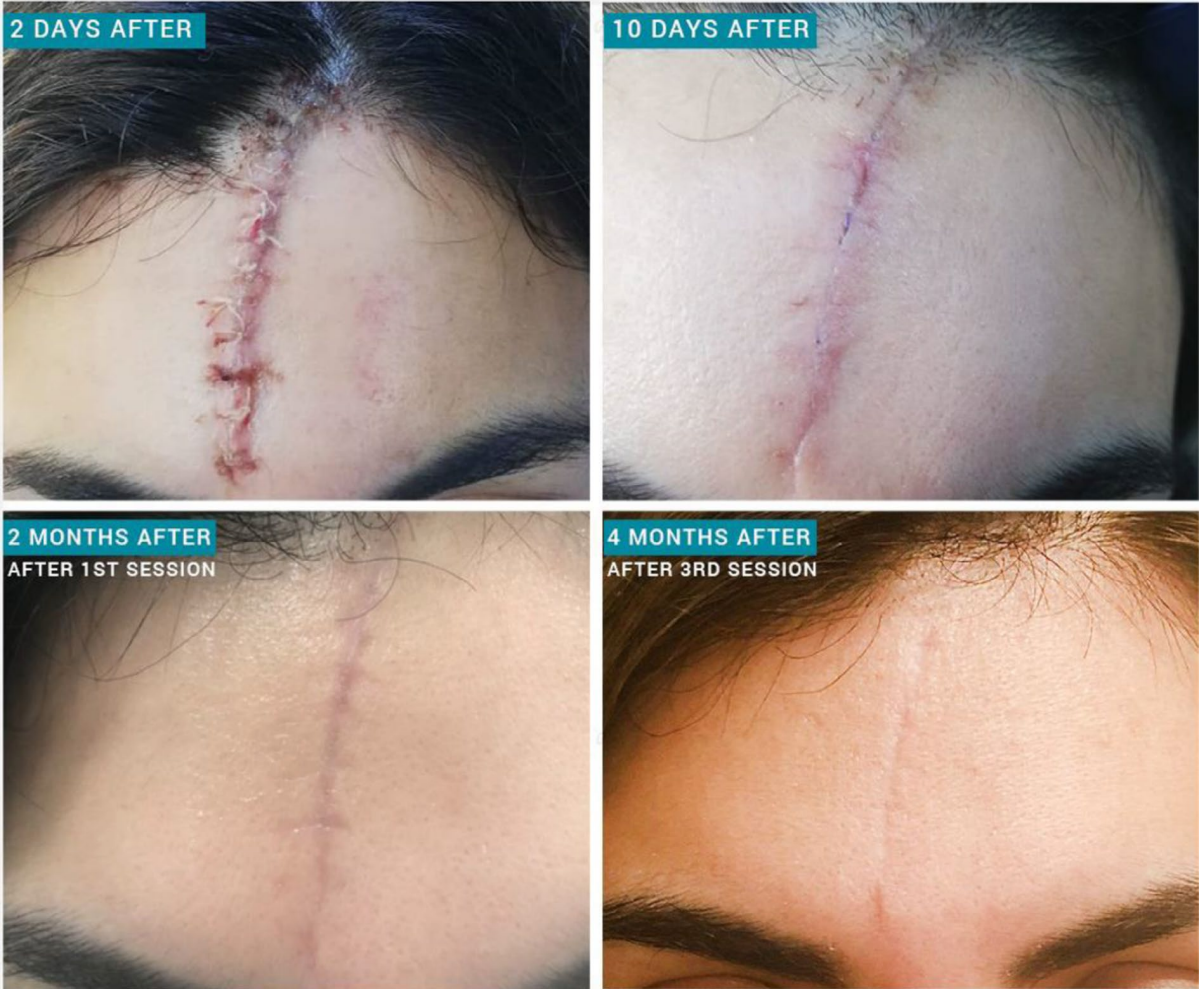
Sequential appearance of the facial scar following the 4th of August Beirut Blast.

A 27‐year‐old healthy woman presented with a 12 cm perpendicular, full‐thickness forehead wound sustained during the Beirut blast on August 4, 2020, as shown in Figure 1.

## 3 Differential Diagnosis, Investigations, and Treatment

Treatment of the forehead with Botulinum toxin A (BTX‐A), as well as perilesional injection of 2 units for every 1 cm around the edges of the wound, was done 2 weeks after the injury. This was followed by 4 sessions of laser treatments separated by a 4‐week interval (Figure 1). Each laser session consisted of 3 steps. The first step consisted of 2 passes by the Nd:YAG laser. The laser parameters were a fluence of 15 J, a spot size of 9 mm, and a pulse duration of 0.6 ms. The second step entailed one pass of total ablative resurfacing with a 2940 nm Er:YAG laser. The laser parameters were a fluence of 2 J, a spot size of 5 mm, and a frequency of 2 Hz. Finally, the third and last step comprised 3 passes of fractional resurfacing with a 2940 nm Er:YAG laser. The laser parameters were a fluence of 30 J, a frequency of 1.4 Hz, and a pulse duration of 200 ms.

## 4 Conclusion and Results (Outcome and Follow‐Up)

**FIGURE 2:**
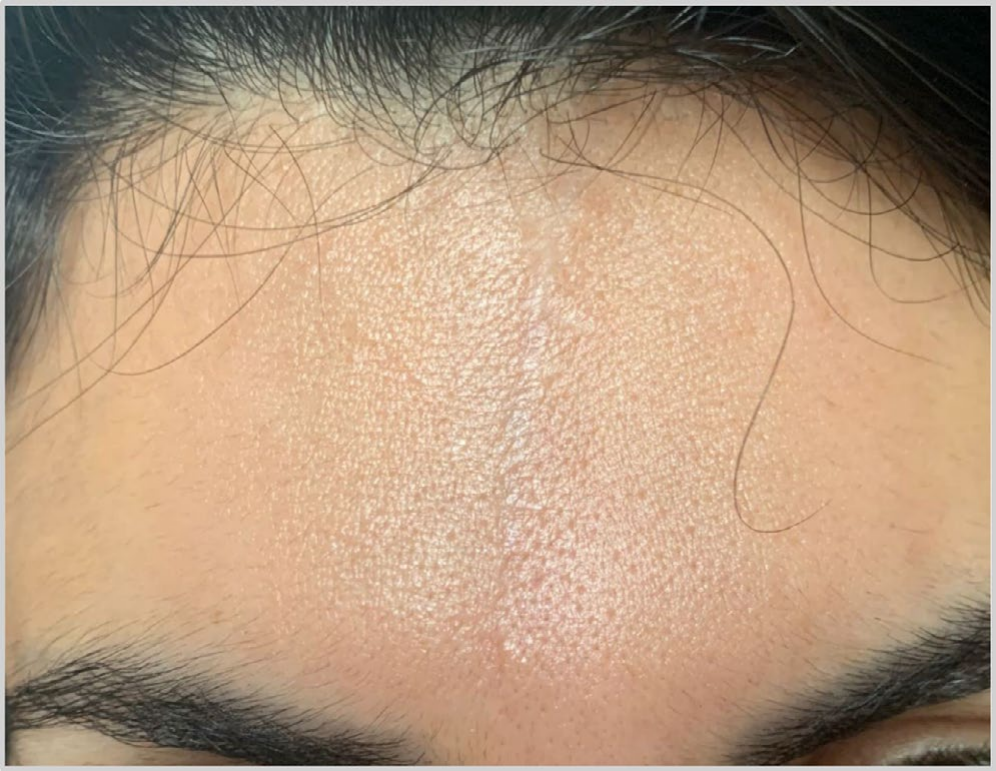
Photo taken a year after the 4th of August Beirut Blast.

Progressive improvement was noted at one‐month interval follow‐ups, with significantly decreased erythema and vascularity, scar width, thickness, and overall appearance (Figure 1). One year later (Figure 2), a noticeable improvement in the scar is noted, a translation of the continuous collagen remodeling months after the last session.

## 5 Discussion

Laser treatment of scars has long been utilized to promote the remodeling of mature scar tissue [3]. Recently, the concept of early laser intervention to reduce scar formation has been investigated in several clinical studies. Laser initiation during the inflammatory phase of wound healing has shown the most benefit, with significant improvement in scar formation [1, 3].

Jang et al. [4] mentioned that the use of a 532‐nm Nd:YAG laser at an early stage of scar formation, within 1–2 weeks after suture removal, resulted in improvements in redness, hyperpigmentation, and hypertrophy of scars within a short period (6 weeks). In another study, Osman et al. [5] demonstrated the equivalent efficacy of Er:YAG and CO_2_ fractional ablative lasers in scar reduction, adding that immature scars respond better than mature scars.

On the other hand, botulinum toxin has been used for decades in the treatment of facial paralysis and cosmetic procedures. It plays a crucial role in scar prevention by relaxing adjacent muscles and minimizing contracture through the inhibition of myofibroblast differentiation and type I collagen synthesis. This was demonstrated in a study by Kim et al. [6] on forehead lacerations, where scar tissue injected with 5 IU/cm of BTX‐A into multiple symmetrical sites at the lower border of the scar resulted in improved aesthetic, functional, and emotional aspects of scar formation in this group.

Additionally, Winayanuwattikun et al. [7] found that post‐mastectomy scar severity decreased, and overall scar appearance improved after BTX‐A injection. The injection was administered 1 cm away from the surgical scar, with 5 units per injection point, totaling 50 units.

Furthermore, the combination of BTX‐A with ablative or nonablative laser treatments may offer superior efficacy compared to monotherapy. Previous studies have shown that both topical and injected forms of BTX‐A can be combined with laser therapy to enhance therapeutic outcomes. Wang et al. studied the injection of BTX‐A before fractional CO_2_ laser treatment and topical growth factors, demonstrating superior efficacy in reducing scars [8].

In our patient, the combination of early perilesional botulinum toxin injection and laser treatments significantly improved scar appearance. We suggest early intervention and repeated treatments for optimal clinical results. Such an outcome may hopefully help erase the memories of the traumatic event that caused the wound in the first place.

## Author Contributions

**Jennifer Akl:** conceptualization, investigation, methodology, writing – original draft, writing – review and editing. **Hamad El Hajj:** conceptualization, investigation, methodology, writing – original draft. **Dima Jeha:** conceptualization, investigation, writing – original draft. **Maya Habre:** conceptualization, investigation, methodology, validation, writing – review and editing.

## Consent

Written informed consent was taken from the patient.

## Conflicts of Interest

The authors declare no conflicts of interest.

## Data Availability

The data used to support the findings of this study are included within the article.
